# Prostatic stromal tumour of uncertain malignant potential: surgical treatment in a young patient

**DOI:** 10.3332/ecancer.2025.1913

**Published:** 2025-05-27

**Authors:** Joaquin Fernandez-Alberti, Alex R Villaalta López, Guillermo Scolari, Alejandro Iotti, Marcelo Featherston

**Affiliations:** 1 Urology Department, Hospital Británico de Buenos Aires, Perdriel 74, CABA, 1280, Buenos Aires, Argentina; 2 Anatomic Pathology Department, Hospital Británico de Buenos Aires, Perdriel 74, CABA, 1280, Buenos Aires, Argentina; a https://orcid.org/0000-0002-1827-7361

**Keywords:** prostate stromal tumour, uncertain malignant potential, radical prostatectomy

## Abstract

Prostate stromal tumour of uncertain malignant potential is a term used to describe a specialised proliferation of stromal cells within the prostate, most tend to be benign, but some can present with local invasion or progress to prostatic stromal sarcoma with distant metastasis. Fortunately, they represent less than 1% of prostate cancers and only a few cases have been described in the literature. We report a case of a 39-year-old male patient who was referred to our centre with this recent diagnosis in the context of acute urinary retention. After an interdisciplinary consideration, a radical prostatectomy was decided for treatment.

## Introduction

Prostatic stromal tumours are rare neoplasms that can be classified as either prostatic stromal sarcoma (PSS) or stromal tumour of uncertain malignant potential (STUMP). These tumours represent an exceptionally uncommon subset of prostatic diseases, accounting for less than 1% of all prostate malignancies [1].

STUMPs are characterised by an atypical and unique stroma proliferation of the prostate. Five patterns have been described: degenerative atypia matrix (most common); a high density of spindle cells; mucus-like spindle cells; phyllodes-like pattern and a newly discovered round cell subtype [2]. Immunohistochemical analysis typically reveals positivity for vimentin, CD34, HF35, desmin, estrogen receptor and progesterone receptor. Notably, cases classified as PSS do not express HF35, smooth muscle actin or desmin [3].

Although STUMPs are generally considered benign, they are classified as neoplastic due to their potential for recurrence, diffuse infiltration of the prostate gland, extension into adjacent structures, and in some cases, progression to PSS with distant metastases [4].

The age at diagnosis varies widely, ranging from 25 to 86 years, with peak incidence occurring in the sixth and seventh decades of life [5]. Clinically, STUMPs may follow an indolent course or present with lower urinary tract symptoms, elevated prostate-specific antigen (PSA) levels, hematuria, abnormal findings on digital rectal examination or rectal obstruction, depending on tumour size and location [6]. While these tumours generally do not exhibit symptoms of aggressive local invasion, certain cases may present with extensive disease progression.

Due to the rarity of STUMPs, no standardised treatment guidelines exist. Management strategies range from active surveillance to surgical resection, depending on tumour behaviour and patient characteristics [7]. Here, we report the case of a young patient with an unusual presentation of acute urinary retention, who underwent radical laparoscopic prostatectomy in 2023 due to a locally aggressive prostatic STUMP.

## Case presentation

A 39-year-old male with no significant personal or family medical history, except for smoking, was referred to our centre with a recent diagnosis of prostatic STUMP in the context of acute urinary retention. The diagnosis was initially established through a transrectal ultrasound-guided prostate biopsy at another institution and was subsequently reviewed and confirmed by our Anatomic Pathology Department. The patient had a body mass index of 22 kg/m² and a good performance status. On physical examination, a functioning cystostomy was observed. A digital rectal examination revealed an enlarged prostate (>100 g) with no additional pathological findings aside from its size. Serum PSA was 4.71 ng/mL and renal function was within normal limits.

**Figure 1.**
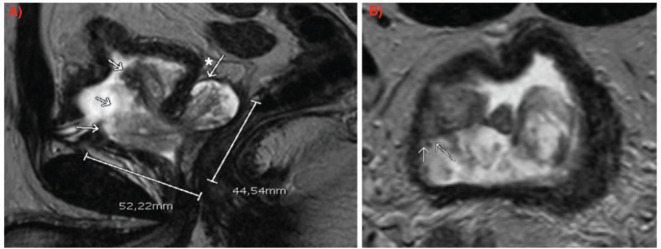
MRI (a): Sagittal slice: arrows indicating intravesical growth of the lesion. The asterisk with the arrow marks the involvement of the left seminal vesicle. (b): Axial slice: arrows indicating tumour contact with bladder wall.

Pelvic magnetic resonance imaging (MRI) demonstrated a large prostatic tumour measuring 45 × 52 × 46 mm, extending from the left seminal vesicle into the bladder lumen (Figure 1). Flexible cystoscopy confirmed intravesical tumour extension without the involvement of the bladder trigone.

The case was discussed in an interdisciplinary tumour board, where radical laparoscopic prostatectomy was recommended as the most appropriate treatment. A transabdominal approach was performed. The seminal vesicles were dissected and the vas deferens were sectioned. This was followed by a Retzius approach to mobilise the bladder and open the endopelvic fascia. After division and ligation of the dorsal vein complex, a cystostomy was performed to remove the intravesical tumour component, and prostatectomy was completed by sectioning the prostatic pedicles using non-absorbable polymer clips. A tension-free vesicourethral anastomosis was performed using two continuous 3-0 absorbable sutures, followed by bladder closure. No pelvic lymph node dissection was performed. The cystostomy was removed and a 16-French urethral Foley catheter was placed for 15 days. The postoperative course was uneventful and the patient was discharged on postoperative day 3.

**Figure 2.**
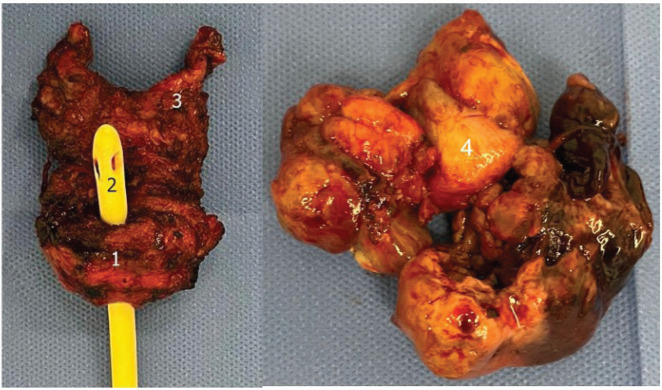
Surgical specimen. 1: Prostate; 2: Urethral foley catheter; 3: Left seminal vesicle; 4: Intravesical extension.

**Figure 3.**
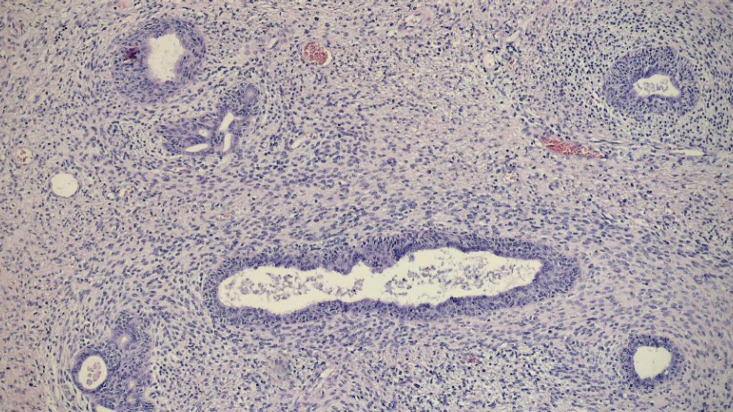
Histopathology (4×): atypical spindle cell proliferation.

Macroscopic examination of the surgical specimen (Figure 2) revealed a 12 × 6 cm tumour with a significant necrotic component, and the prostate weighed 49.6 g. Histological analysis confirmed a STUMP, characterised by a proliferation of spindle-shaped stromal cells with mild nuclear atypia (phyllodes-like pattern) and a mitotic count of 3 mitoses per 10 high-power fields (Figure 3). Immunohistochemical staining was performed on 3-micron histological sections using an automated system (Benchmark XT, ULTRA) in accordance with the manufacturer’s guidelines. Atypical cells were positive for vimentin, actin and CD34, while desmin staining was negative. Based on histopathological and immunohistochemical findings, combined with the patient’s clinical presentation, a diagnosis of prostatic STUMP was confirmed.

Regarding functional outcomes, the patient achieved full urinary continence within 30 days postoperatively. After 14 months of pelvic floor physiotherapy and phosphodiesterase type 5 inhibitor therapy, he regained the ability to achieve penetrative intercourse. PSA levels have remained undetectable, and 12-month follow-up imaging, including multiparametric prostate MRI and thoracic computed tomography (CT) scan, showed no evidence of recurrence.

## Discussion

STUMPs were first described by Gaudin *et al* [3] in 1998 in a series of 22 cases characterised by specific histological and immunohistochemical features that differentiate them from PSSs. These tumours represent rare proliferative lesions of the prostate derived from specialised stromal cells and exhibit significant biological variability. While some cases cause only mild local morbidity, others may behave more aggressively, infiltrating adjacent tissues or even progressing to PSS with distant metastases [4, 8].

Diagnosis is typically established through core biopsy, although some cases are incidentally identified in specimens obtained from transurethral resection of the prostate or radical prostatectomy performed for suspected adenocarcinoma. As previously described, five histological subtypes of STUMP have been identified [2], and immunohistochemical analysis is essential for an accurate diagnosis. Given the rarity of this entity and its histological overlap with sarcomas, we strongly recommend that biopsy specimens be reviewed by experienced pathologists at high-volume centres specialising in urological oncology. Correct differentiation from sarcomas is crucial, as it significantly impacts treatment decisions and prognosis. Additionally, before determining a treatment strategy, thorough staging should be performed, including pelvic MRI and cystoscopy to assess local extension, as well as thoracic CT to rule out distant disease.

Colombo *et al* [9] reported fewer than 15 cases of prostatic STUMP in patients younger than 40 years. Due to the rarity of this tumour, there is no established consensus on the optimal treatment approach. When considering definitive surgical resection, several factors must be taken into account, including patient age, tumour extension, symptomatology and institutional experience with rare urological diseases. In our case, an interdisciplinary tumour board—including urologists, oncologists, radiologists and pathologists—determined that radical laparoscopic prostatectomy was the most appropriate treatment, given the patient’s young age, acute presentation and localised disease.

When determining the most appropriate surgical approach, we opted for radical prostatectomy instead of cystoprostatectomy. Preoperative imaging and intraoperative assessment confirmed intravesical tumour extension without significant bladder wall involvement, particularly sparing the trigone. Given the patient’s young age and the goal of preserving bladder function while minimising surgical morbidity, radical prostatectomy was considered the most suitable treatment option. Pelvic lymph node dissection was not performed since both preoperative imaging and intraoperative assessment showed no evidence of lymph node involvement, and given the localised nature of STUMP, lymphadenectomy is not routinely indicated. Furthermore, if PSS is a potential differential diagnosis, its primary route of dissemination is hematogenous rather than lymphatic.

Dokubo *et al* [10] described two cases of STUMP managed with robot-assisted radical prostatectomy, reporting favourable oncological and functional outcomes after nearly 5 years of follow-up. Although our follow-up period is shorter, our patient has demonstrated similar results, further supporting radical prostatectomy as a valid treatment option for this type of presentation. However, given the risk of local recurrence and potential progression to PSS with metastases to the bones and lungs, close clinical and imaging follow-up is essential. Although PSA is the standard follow-up marker for prostate adenocarcinoma, its utility in STUMP is less well established due to the typically low PSA production by these tumours. In our case, the preoperative PSA was measurable and postoperative levels remained undetectable, yet we acknowledge that PSA monitoring should be interpreted with caution in STUMP cases.

## Conclusion

Due to its rarity and unpredictable clinical behaviour, there is no established consensus on the optimal management of STUMPs. A thorough diagnostic workup, including assessment of local and distant disease extension, is essential for guiding treatment decisions. In young patients with localised disease and good performance status, surgical excision should be strongly considered to reduce the risk of recurrence and potential malignant transformation. As more cases are reported and long-term follow-up data become available, a better understanding of STUMP will help refine treatment strategies and improve patient outcomes.

## Conflicts of interest

None declared.

## Funding

The authors have not declared a specific grant for this research from any funding agency in the public, commercial or not-for-profit sectors.

## Author contributions

All authors contributed to the study conception and design. Material preparation, data collection and analysis was performed by Dr Alex R Villaalta López. The first draft of the manuscript was written by Drs Joaquín Fernandez-Alberti and Alex R Villaalta López and all authors commented on previous versions of the manuscript. All authors read and approved the final manuscript.

## References

[1] Nagar M, Epstein JI (2011). Epithelial proliferations in prostatic stromal tumors of uncertain malignant potential (STUMP). Am J Surg Pathol.

[2] Sadimin ET, Epstein JI (2016). Round cell pattern of prostatic stromal tumor of uncertain malignant potential: a subtle newly recognized variant. Hum Pathol.

[3] Gaudin PB, Rosai J, Epstein JI (1998). Sarcomas and related proliferative lesions of specialized prostatic stroma: a clinicopathologic study of 22 cases. Am J Surg Pathol.

[4] Kabarriti AE, Guzzo TJ, Wein AJ (2014). Prostate stromal tumor of uncertain malignant potential: case report with 5-year follow-up. Urol Case Rep.

[5] Muglia VF, Saber G, Maggioni G (2011). MRI findings of prostate stromal tumour of uncertain malignant potential: a case report. Br J Radiol.

[6] Addesso M, Caputo A, Zeppa P (2023). Prostatic stromal sarcoma: report of a rare case in a young male and review of the literature. Int J Surg Case Rep.

[7] Murer LM, Talmon GA (2014). Stromal tumor of uncertain malignant potential of the prostate. Arch Pathol Lab Med.

[8] Herawi M, Epstein JI (2006). Specialized stromal tumors of the prostate: a clinicopathologic study of 50 cases. Am J Surg Pathol.

[9] Colombo P, Ceresoli GL, Boiocchi L (2010). Prostatic stromal tumor with fatal outcome in a young man: histopathological and immunohistochemical case presentation. Rare Tumors.

[10] Dokubo II, Tay LJ, Rutigliani L (2023). Prostatic stromal tumour of uncertain malignant potential treated with robotic-assisted radical prostatectomy: medium-term oncological and functional outcome of two cases. Ann R Coll Surg Engl.

